# Stress–induced elemental retention in an ectothermic vertebrate

**DOI:** 10.1242/bio.062237

**Published:** 2025-10-02

**Authors:** Avik Banerjee, Maria Thaker

**Affiliations:** Centre for Ecological Sciences, Indian Institute of Science, Bengaluru, India

**Keywords:** carbon, nitrogen, nutrients, reptile, corticosterone, glucose

## Abstract

Physiological stress responses are energy intensive. Animals can meet their stress-induced energetic demands by altering foraging or selectively retaining ingested nutrients, although the latter is poorly studied. We tested the effects of elevated stress on elemental retention in *Psammophilus dorsalis*. Adult lizards of both sexes were allotted to either a stressed group (daily constraint) or control group for 10 days. We measured baseline corticosterone, glucose, and triglyceride levels of lizards at the beginning and end of the experiment, as well as the total amounts of carbon and nitrogen retained based on the ingested and egested content during the treatment phase. Both control and stressed groups had higher corticosterone levels at the end of the experiment, with stressed group males showing the greatest increase. Glucose and triglyceride levels were variable. Contrary to expectation, lizards from both treatments retained similar amounts of carbon and nitrogen during the experiment phase. Our results do not show support for changes in elemental retention under stressful conditions, although the stress of captivity itself could have masked the potential effect on elemental retention. Our study highlights the need to test elemental retention as a potential strategy to meet stress-induced energetic demands when foraging opportunities are limited.

## Introduction

Ecological challenges are known to elicit physiological and behavioural responses in animals, which when mediated by glucocorticoid hormones, is widely characterized as the “stress response” (Wingfield, 2013; Sapolsky et al., 2000). Glucocorticoids are highly conserved across vertebrate taxa (Schoenle et al., 2021; Bonier et al., 2009; Romero et al., 2009) and act as essential orchestrators of the coping response of individuals by mobilizing energy to essential organs and supporting survival functions that maintain allostasis (Wingfield et al., 2017; Crespi et al., 2013; Bonier et al., 2009; Sapolsky et al., 2000). This is accomplished by re-routing nutrients, especially carbon-rich macronutrients (such as carbohydrates and lipids), to support survival functions (Hawlena & Schmitz, 2010; Bonier et al., 2009; Landys et al., 2006). If the challenge is prolonged and requires sustained energy supply, nitrogen-rich macronutrients such as proteins, which are typically utilized for growth and reproduction, are temporarily shunted towards energy production. Excess amounts of nitrogen-rich proteins that cannot be stored or immediately utilized are eliminated via faeces to reduce the costs of storing excess non-essential nutrients during the challenging period (Hawlena & Schmitz, 2010). Therefore, when animals face stressful periods in the wild, they are expected to actively forage for food resources that meet the elevated nutrient demands of the physiological stress response (Banerjee et al., 2025; Felton et al., 2021; Lambert & Rothman, 2015; Simpson & Raubenheimer, 2012; Raubenheimer & Simpson, 2003). However, when animals cannot meet their optimal nutrient needs in the wild through foraging, they should switch to post-ingestive strategies such as selective retention of ingested nutrients to meet these stress-induced energetic requirements.

Multiple studies have provided both functional and mechanistic descriptions of post-ingestive regulation of nutrients. For example, the grasshopper, *Melanoplus differentialis*, (Yang & Joern, 1994) and red-winged blackbirds, *Agelaius phoeniceus*, (Brugger, 1991) are known to increase their gut size to enhance nutrient absorption in response to reduced food quality. The locust, *Locusta migratoria*, modulates gut enzymes to obtain specific macronutrients from their diet (Clissold et al., 2010). Ectothermic vertebrates, especially reptiles, which are generally known to have higher digestive efficiency compared to similar sized endothermic vertebrates (Wehrle & German, 2023), have been found to increase gut retention times in response to increasing temperatures (Plasman et al., 2019; McConnachie & Alexander, 2004; Wehrle & German, 2023) or decreasing nutritional quality of food (van Marken Lichtenbelt, 1992). Therefore, post-ingestive nutrient regulation as an adaptive response to meet nutritional homeostasis (i.e., optimal intakes of nutrients) is widespread across several taxa (Raubenheimer & Simpson, 1997). Whether animals employ selective retention of nutrients during stressful conditions, however, remains relatively unexplored.

Our aim was to explore the link between physiological stress responses and nutrient (via elemental) retention in ectothermic vertebrates. Using the model lizard species, *Psammophilus dorsalis*, we conducted a stress manipulation experiment in the lab wherein wild-caught adult lizards were subjected to either a control (undisturbed) or a constraint-stress treatment. We continuously measured the amounts of food ingested and the mass of faecal matter egested by each lizard, and calculated the retention of carbon and nitrogen in both treatment groups. Carbon and nitrogen levels are good proxies for carbohydrates and proteins in arthropods (Reeves et al., 2021), and enables us to use the same units of measurement for ingested and egested content. We quantified the physiological state of the animal by measuring baseline corticosterone levels before and after treatment, with the prediction that corticosterone levels would be higher in the stressed treatment group compared to the control group. We also quantified glucose (primary energy metabolite) and triglyceride (stored lipid reserves) levels in plasma, which are energy metabolites expected to be circulating and fuelling the stress-induced energetic needs of these individuals (Price, 2017). We predicted that glucose and triglycerides levels would be elevated in individuals from the stressed treatment group (Banerjee et al., 2025; Millanes et al., 2024; Jimeno & Verhulst, 2023; Remage-Healey & Romero, 2001). Further, we predicted that stressed lizards would retain more carbon and eliminate more nitrogen compared to the control group to meet the higher energetic needs induced by elevated physiological stress. Our study explores post-ingestive elemental retention as an adaptive compensatory strategy to fulfil increased energetic needs of ectothermic vertebrates in stressful conditions when pre-ingestive nutrient acquisition is limited.

## Materials and Methods

### Model organism

*Psammophilus dorsalis* is a sexually dimorphic agamid lizard found in semi-arid landscapes of peninsular India. Males are larger than females in this species. They are most active between February to September with the breeding season lasting between March to August (Radder et al., 2005). They are opportunistically insectivorous, predominantly feeding on ants (Banerjee et al., 2025; Balakrishna et al., 2016).

### Capture and handling

Adult lizards of both sexes (N=23, males=11, females=12) were collected from the rocky hills of Avathi, Bengaluru, India towards the end of their breeding season in the months of August and September of 2021. Lizards were captured by lassoing and transported to the lab. Lizards were maintained in glass terraria (60cm x 40 cm x 40 cm) lined with tissue paper substratum and provided with a small rock perch and a Petri dish with *ad libitum* water. Terraria were housed in a dedicated housing room maintained at ambient room temperature (~28° C) with 12 hours light/dark cycle. A basking light (100 W incandescent bulb) fitted ~30cm above each tank was turned on three times during the day for 1-hour period each.

Lizards were first allowed to acclimate to the laboratory conditions for 5 days, which ensured that they started feeding on live mealworms. During the acclimation period, all lizards were provided with 4 live mealworms/day. Lizards (n = 1 male and 1 female) that did not eat the live mealworms during the acclimation phase were not included in the subsequent manipulative experiment (detailed below). After the acclimation period, lizards (N=21, 10 males and 11 females) were subjected to the manipulative experiment which lasted for 10 days. Body mass (in g) and snout-vent length (SVL, in mm) were recorded for all individuals at the beginning and the end of the experiment using a digital weighing scale and a ruler respectively. At the end of the experiment, lizards were marked with non-toxic ink on their ventral surface to prevent recapture before they were released at their site of capture.

### Manipulative experiment

**Fig. 1:**
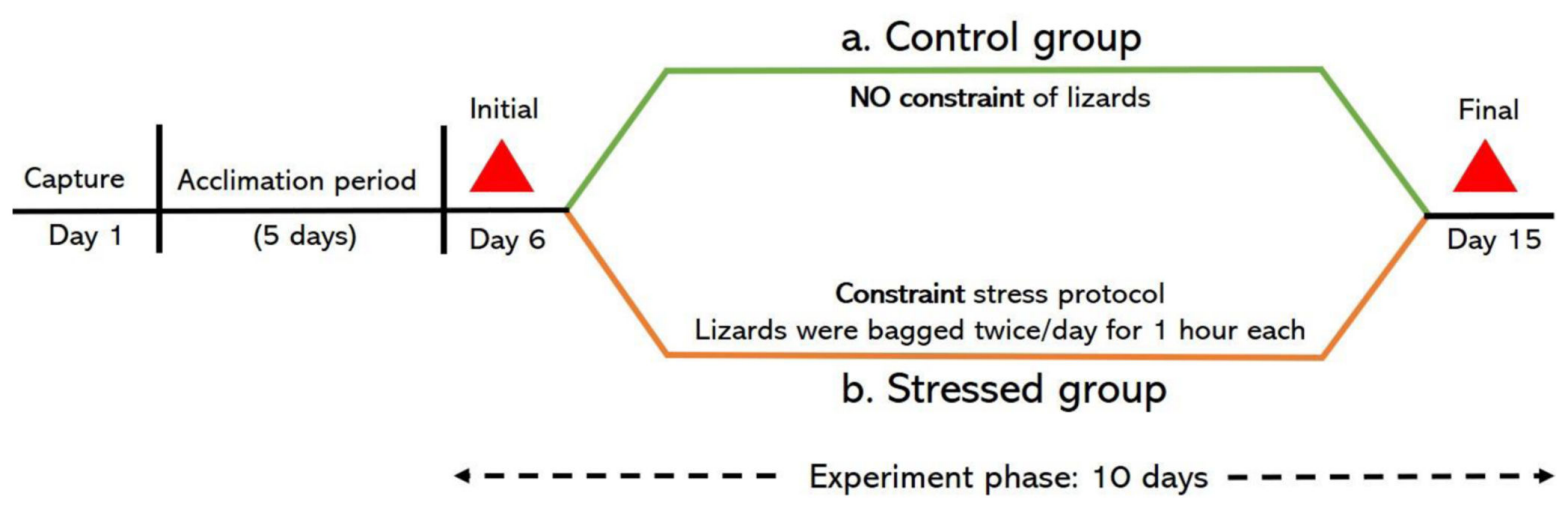
Schematic of the experimental regimen. Lizards were captured from the wild and allowed to acclimate in the lab for 5 days before being exposed to either a control (green solid line) or stressed (orange solid line) treatment which lasted for 10 days (black dashed double arrow). Blood samples, denoted by red triangles, were obtained on the initial (day 6) and final (day 15) day of experiment phase.

Lizards were assigned to a control (n=5 males, SVL (mean ± s.d.) = 117.4 ± 5.7 mm, and 6 females, 87 ± 6.1 mm) or stressed (n=5 males, 119 ± 4.3 mm, and 5 females, 88.6 ± 4.2 mm) treatment group. Assignment to treatment groups was such that the body size of lizards did not significantly differ between the treatment groups for both males (unpaired t-test, t=-0.502, P=0.6) and females (t=-0.494, P=0.6). The manipulative experiment ran for 10 days including blood sampling on the first and last day. For the stressed treatment group, lizards of both sexes were subjected to a constraint stress protocol for 8 days with no restraint on the days of blood sampling. The constraint stress protocol involved putting the lizard in a cotton bag for a 1 hour duration, twice a day (French et al., 2006). The time of bagging the lizards was selected at random to avoid learning or habituation. An inter-bagging interval of a minimum 2 hours was maintained. Lizards in the control treatment group were not subjected to this constraint stress protocol during the experiment phase. All other conditions were same for both the treatment groups (see Figure 1).

Regardless of treatment group, males were provided with 8 mealworms per day and females with 6 mealworms per day throughout the experiment phase. Differences in the number of mealworms provided for each sex was because males are larger than females, and can ingest more than females when provided with *ad libitum* food. Daily intake of food was held constant throughout the experiment to control for intake-based variation (Wehrle & German, 2023). Food was presented at two different times of the day to replicate the dual activity period reported for this species (Radder et al., 2005) and to avoid the risk of lizards not feeding as a consequence of the stress handling. Mealworms provided as food were weighed before and after the feeding opportunity to calculate the amount of food ingested. Additionally, whole mealworm samples were also collected, freeze-killed and stored in 15mL vials at -20°C to estimate their carbon and nitrogen content (see methods below). Faecal samples of lizards were also collected throughout the experiment phase and stored in 5mL vials at -20°C for similar elemental composition analysis (see methods below). We also quantified the proportion of urates eliminated, which was calculated as the total weight of urates (in g) divided by the total faecal weight (in g) excreted.

### Hormone and metabolite

We collected a blood sample (~40-100 μL) from the retro-orbital sinus of lizards (following Thaker et al., 2009) on the first day and the last day of the experiment phase. Blood samples were collected using heparinized capillary tubes within 3-min of capture during 0600-0700 hours to estimate baseline levels of hormones and metabolites (Tylan et al., 2020). We immediately measured the circulating glucose levels (mg/dL) from the blood samples using a hand-held Glucometer (Contour Next ONE, Bayer/Ascensia) (Sparkman et al., 2018). The remaining blood sample was centrifuged, and the separated plasma was stored in -80°C until further analyses.

We quantified the baseline corticosterone levels (ng/mL) from the plasma samples using a standard enzyme immune-assay kit (K014-H5, Arbor Assays) that had been previously optimised for this species (Batabyal & Thaker, 2019). In the assays, we used 4 μL plasma volume with a dilution ratio of 1:100. All the samples were run in triplicates along with a lab standard of known concentration in duplicate in each assay. The intra-assay coefficient of variation (CV) ranged between 1.36 - 14.6 (mean ± s.d.= 6.02 ± 0.1) and the inter-assay CV was 8.55 ± 2.35 (mean ± s.e.m.).

Triglyceride levels (mg/dL) were also quantified from the plasma samples using standard triglyceride calorimetric assay kits (MAK266, Sigma Aldrich). Calorimetric assays were run using 6 μL of plasma sample in triplicate involving a lipase and enzyme treatment to produce the colour. Each assay plate (96-well flat bottom) was run with a standard curve based on a serial dilution of triglyceride standard provided with the kit and the plate was read at 570 nm. R-squared values for all plates against the standard curve were greater than 0.98 with the intra-assay CV ranging between 1.65 – 12.62 (mean ± s.d.= 2.79 ± 0.02).

### Elemental analyses

For the elemental analysis of ingested and egested content by lizards, mealworm and faecal samples were first dried in a hot-air oven at 60°C for a minimum of 12 hours. Dry weights were recorded. Additionally, for faecal samples, we also separately recorded the dry weight of the urate part of faeces since urates represent the bulk of nitrogenous wastes in reptiles. All the samples were then manually crushed using a mortar and pestle to a dry homogenous mixture. This mixture was packed in tin foil and analysed using a CHNS analyser (Elementar Analyse Systeme GmbH, Germany) to estimate the carbon (%) and nitrogen (%) content. The mean carbon and nitrogen (%) estimates of mealworms were multiplied by the weight of all mealworms ingested to calculate the total elemental intake of lizards during the experiment phase (as per Banerjee et al., 2025). Similarly, for egested amounts, carbon (%) and nitrogen (%) values for each faecal sample were multiplied with the faecal weight and summed over the experiment phase. Using the ingested and egested amounts, we calculated the elemental retention efficiency, or ratio of elements retained to elements consumed using the following equation (1);

(1) $$\text{Retention}\;\text{efficiency}\;\text{of}(\text{C}\;\text{or}\;\text{N})\;=\frac{\text{Weight}\;\text{of}\;(\text{C}\;\text{or}\;\text{N})\;\text{ingested}\;(\text{in}\;\text{g})\;-\;\text{Weight}\;\text{of}\;(\text{C}\;\text{or}\;\text{N})\;\text{egested}\;\;(\text{in}\;\text{g})}{\text{Weight}\;\text{of}\;(\text{C}\;\text{or}\;\text{N})\;\text{ingested}\;(\text{in}\;\text{g})},$$

### Statistical analyses

All the statistical analyses were performed in R (version 4.4.0). For corticosterone, glucose, and triglyceride levels, we used separate generalized linear mixed effect models (GLMM), with treatment group (i.e., control or stressed) and stage of experiment when the blood sample was extracted (i.e., start or end of treatment) as predictors with a 2-way interaction. Animal ID was included as a random variable to account for repeated measures of the same individuals. Models followed a Gamma distribution for positive non-normal continuous data and were run using the R package *lme4* (Bates et al., 2015). Normality was checked using Shapiro-Wilk test in all cases (p<0.05). We ran separate models for males and females for these response variables because the size difference between sexes is known to affect physiological parameters (Banerjee et al., 2025). Since our sample size was limited, we bootstrapped (n=100) our model coefficients and compared the confidence intervals (represented as 95% CI) with the model outputs from the GLMM. Continuous positive and negative confidence intervals indicated the statistical precision of significance.

We compared total carbon and nitrogen ingested (g) and egested (g) by lizards during the experiment using separate GLM models following a Gamma distribution with treatment group (control or stressed) and sex (male or female), as predictors in a 2-way interaction using the R package *lme4* (Bates et al., 2015). Similarly, we determined the effects on elemental retention efficiency of carbon and nitrogen using GLM models (following Beta regression for continuous proportion data), with treatment group and sex as predictors in a 2-way interaction using the R package *glmmTMB* (Brooks et al., 2017).

Finally, to determine if the mass of lizards changed due to the experimental treatment, we ran a GLM (with Gaussian distribution) with body mass change of lizards (i.e., mass at the end of treatment – mass at the start of treatment) as a function of treatment group and sex with an interaction effect. Post-hoc pairwise comparisons were done when relevant using the R package ‘*emmeans*’ (Lenth et al., 2019) with Tukey's HSD corrections (for detailed model outputs, see Supplementary material, Table S1, S2, S3, and, S4).

### Ethics statement

The Animal Ethics Committee of the Indian Institute of Science has approved all the protocols used in the study (Permit number: CAF/Ethics/867/2021). Sample sizes for the study were determined based on what was approved by this committee. Collection permits were not required since *P. dorsalis* is not protected under the Schedules of the Indian Wildlife (Protection) Act, 1972.

## Results

### Hormones and metabolites

**Fig. 2:**
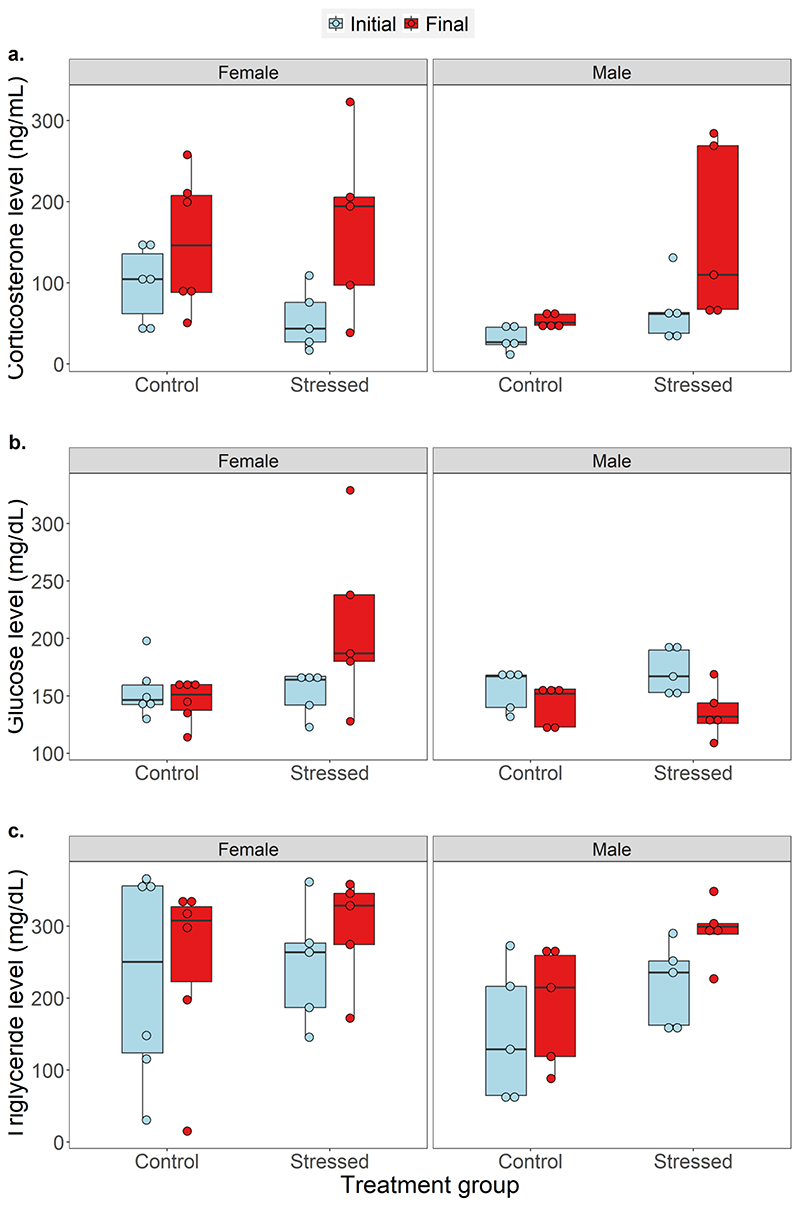
Initial (blue) and final (red) levels of a. baseline corticosterone, b. glucose, and, c. triglyceride of *Psammophilus dorsalis* from control (n= 6 females and 5 males) and stressed (n= 5 females and 5 males) treatment groups. For each response variable, comparisons were done for males and females separately using a GLMM (following gamma) with treatment group and stage of experiment in a two-way interaction including animal ID as a random variable.

There was a significant interaction effect of treatment group (control vs stressed) and stage of experiment (start vs end of treatment) for corticosterone levels in females (t= -2.58, p= 0.01, 95% CI= -1.587, -0.091) but not in males (t= -0.7, p= 0.5, 95% CI= -1.052, 0.789). Pairwise comparisons showed that final corticosterone levels of females were significantly higher than initial levels in both the control (Z=2.05, p=0.04) and stressed (Z=5.191, p<0.01) treatment groups. In contrast, for males, treatment group (t= 2.992, p< 0.01, 95% CI= 0.349, 1.477) and stage of experiment (t= -2.293, p= 0.02, 95% CI= -1.038, -0.266) had independent effects on baseline corticosterone levels. Final corticosterone levels of males were significantly higher than initial levels for both the control (Z=2.293, p=0.02) and stressed (Z=3.183, p<0.01) treatment groups. Moreover, in males alone, final corticosterone levels were significantly higher in the stressed group compared to the control group (t=-2.992, p<0.01, Figure 2a).

Unlike corticosterone, there was a significant interaction effect of treatment group and stage of experiment on circulating glucose levels for both females (t= -2.933, p<0.01, 95% CI= -0.697, -0.107) and males (t= 2.381, p= 0.02, 95% CI= 0.022, 0.315). Initial and final glucose levels did not differ for the female control group (Z= -0.674, p= 0.5). But for the female stressed group, final glucose levels were significantly higher than initial levels (Z= 3.354, p<0.01). In contrast, males showed an opposite pattern, wherein final glucose levels were significantly lower than initial levels for both the control (Z= -2.068, p= 0.04) and stressed (Z= -5.435, p<0.01) treatment groups (Figure 2b).

For triglyceride levels, we found no interaction effect of treatment group and stage of experiment for both females (t= -0.518, p= 0.6, 95% CI= -0.861, 0.675) and males (t= 0.131, p= 0.9, 95% CI= -0.31, 0.372). Stage of experiment had a significant main effect on triglyceride levels for males but not females. Compared to initial levels, final triglyceride levels of males were significantly higher for both the control (Z= 3.147, p<0.01) and the stressed (Z= 2.968, p<0.01, Figure 2c) treatment groups. All other pairwise comparisons were not statistically significant (see Supplementary material, Figure S1 for within-individual variations). ‘Animal ID’ had a negligible effect in all the models (GLMM random effect; s.d.<0.5, variance<0.4).

### Food ingested, faecal egested and elemental retention

The elemental content of mealworms provided as food during the experimental phase was estimated to contain 8.0 ± 0.16% (mean ± s.e.m.) of nitrogen and 54.31 ± 0.88% of carbon. Lizards of both treatment groups ate nearly all mealworms provided during the experimental phase, and thus the total ingested amounts of carbon and nitrogen in terms of weight (g) were not significantly different for both stressed and control treatment groups of males and females (see Table 1 and Supplementary material, Figure S2). There was no significant interaction effect between treatment and sex for ingested amounts of carbon and nitrogen (t=0.174, p=0.9).

There was also no significant interaction effect between treatment and sex for egested amounts of carbon (t=-0.04, p=0.9) and nitrogen (t=0.297, p=0.8, see Supplementary material, Table S1). We found no significant difference in egested amounts of carbon (g) and nitrogen (g) over the experiment period for both males and females (see Table 1 and Supplementary material, Figure S2). The proportion of urates eliminated during the experimental phase by the control groups (mean ± s.e.m.; males=0.61 ± 0.02, and, females=0.59 ± 0.02) were also similar to those eliminated by the stressed treatment groups (males=0.62 ± 0.01, and, females=0.55 ± 0.02).

**Fig. 3:**
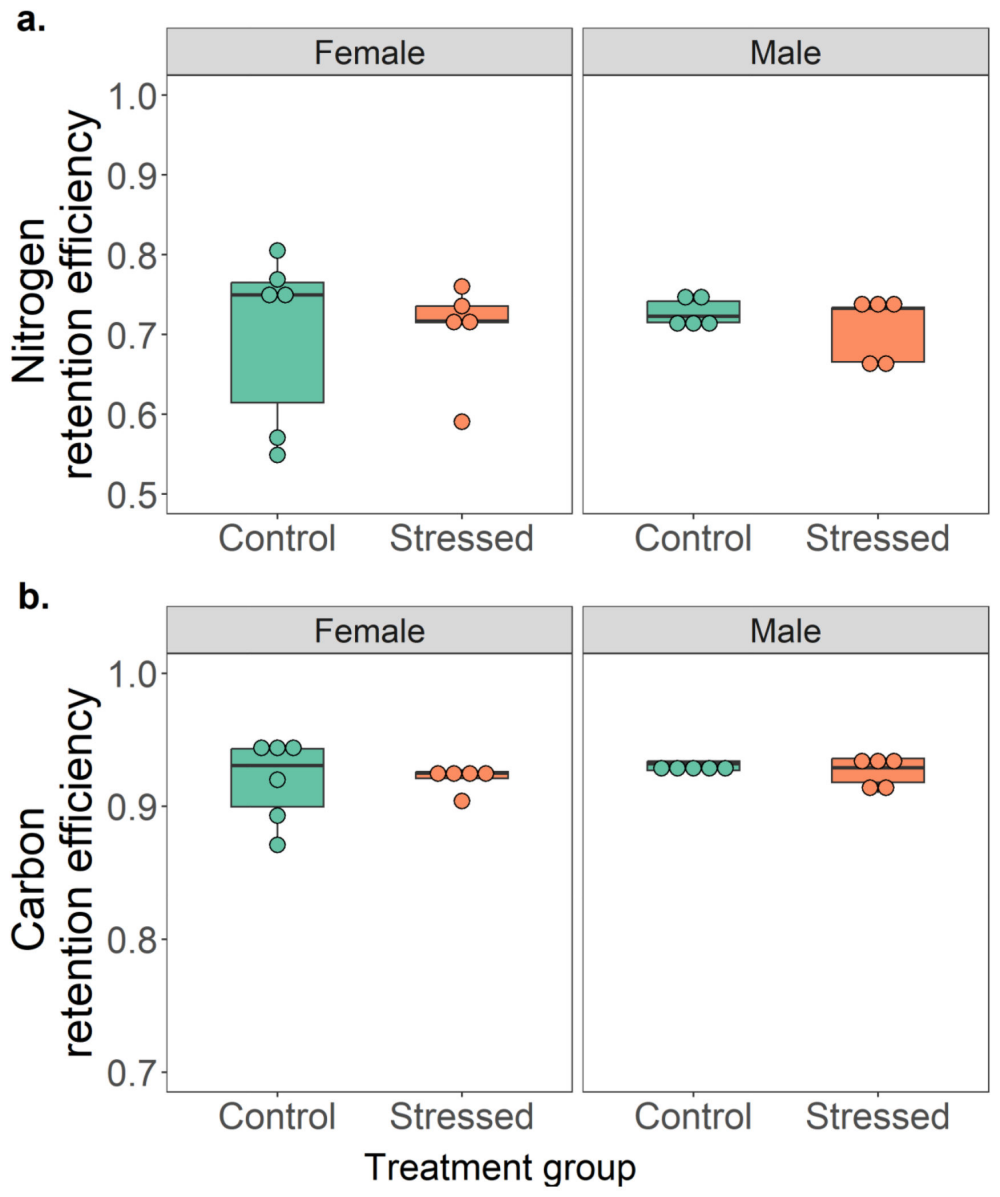
Retention efficiency of a. Nitrogen, and, b. Carbon, of Psammophilus dorsalis from control (n= 6 females and 5 males) and stressed (n= 5 females and 5 males) treatment groups. For each response variable, comparisons were done using a GLM (following beta regression) with treatment group and sex in a two-way interaction. No significant differences between treatments and sex were found (p > 0.05).

Further, we found that there was no significant interaction effect between treatment and sex for retention efficiency of carbon (t=-0.06, p=0.9) and nitrogen (t=-0.341, p=0.7). However, lizards retained a higher amount of carbon than nitrogen, such that males and females of both control and stressed treatment groups retained approximately 1.3 times more carbon (mean ± s.e.m. = 0.925 ± 0.006 or approximately 93% of ingested amounts) than nitrogen (0.71 ± 0.007 or approximately 71% of ingested amounts, see Table 1 and Figure 3).

Finally, treatment group and sex did not have a significant interaction effect (t=-0.933, p=0.4) on the body mass change of lizards. Although, lizards in both treatment groups showed lower body mass at the end of the experiment compared to the beginning, these were not statistically significant (see Supplementary material, Figure S3).

## Discussion

Animals frequently encounter ecological challenges in the wild that are typically mediated by a physiological stress response (Hawlena & Schmitz, 2010; Nelson, 2005; Sapolsky et al., 2000). Animals are expected to meet the increased energy demands of this stress response by actively foraging for food resources that enable rapid conversions to useable energy. However, when pre-ingestive strategies for resource acquisition are limited by ecological constrains, such as resource uncertainty, competition, predation risk, as well as seasonal or climatic factors (Teichroeb et al., 2024; Des Roches et al., 2022; Ferretti et al., 2019; Huey & Kingsolver, 2019), animals are expected to switch to post-ingestive regulation of ingested nutrients, which could be a vital adaptive response for optimizing energy usage and maintaining allostasis (Simpson & Raubenheimer, 2012; Bonier et al., 2009; Landys et al., 2006). We tested the potential link between physiological stress and elemental retention in the ectothermic vertebrate, *Psammophilus dorsalis*. We expected lizards that were chronically stressed to retain more carbon and eliminate more nitrogen to meet their stress-induced energetic demands. Contrary to our expectation, we found that lizards from both control and stress treatment groups showed similar patterns of post-ingestive elemental retention. Although inconsistent with our predictions, these results are still intriguing as it highlights the resilience of animals under chronic stress.

We hypothesized that lizards of both sexes that experienced a daily constraint protocol (by being held in a cloth bag) would exhibit increased levels of corticosterone compared to control group lizards that were undisturbed. We found that corticosterone levels, which is primarily the metabolic hormone mediating stress responses in reptiles, was higher for both the stress and control treatment groups at the end of the experiment. There could be multiple reasons to explain this observed pattern. Lizards in captivity have restricted movement and foraging options, and are exposed to human interference every day, during feeding or whenever a researcher enters the housing facility, therefore captivity itself could have elevated stress responses in both the treatment groups (Hawlena & Pérez-Mellado, 2009; Morgan & Tromborg, 2007). Also, collection of blood sample at the start of the experiment from the control and stressed treatment lizards could have induced unavoidable stress that was long lasting (Langkilde & Shine, 2006). It is also possible that the stress-inducing protocol caused initial spikes in corticosterone levels that were subsequently dampened by the negative feedback mechanisms (Bonier et al., 2009). Thus, even though lizards in the control group were not exposed to an additional stress-inducing protocol, above factors could have resulted in similar elevated baseline corticosterone levels for both the treatment groups. Nonetheless, we found that the daily constraint protocol for the stressed treatment group resulted in a significant increase in baseline corticosterone levels in males and not females. Unlike males, there is evidence that females can supress corticosterone responses, especially during the breeding season as a mechanism to avoid transferring excess corticosterone to developing eggs (Ensminger et al., 2018; Saino et al., 2005). Although we conducted this experiment towards the end of their breeding season and females were not visibly gravid when collected, we cannot rule out the possibility of breeding-related hormone regulation by females. In contrast, testosterone-regulated reproductive functions in males during the breeding season, which involve territory defence, increased competition and elaborate courtship behaviours often favour higher levels of glucocorticoids to meet the associated energetic demands (Eikenaar et al., 2012). These sex-differences in stress-responsiveness may explain why males and not females exhibited elevated corticosterone responses to additional stress exposure.

Although, glucose is a primary energy metabolite known to modulate energetic requirements, circulating glucose levels are highly context dependent and their relationship with corticosterone shows wide variation across studies depending on the species, time of day, nutritional state, and life history traits (Banerjee et al., 2025; Millanes et al., 2024; Neuman-Lee et al., 2019; Walsberg, 2003; Remage-Healey & Romero, 2001). Females in the stressed group showed elevated glucose levels at the end of experiment compared to beginning, unlike females in the control group, whose glucose levels stayed similar. Increased glucose levels in stressed females reflects the increased usage of energy in response to additional daily constraint stress treatment (Remage-Healey & Romero, 2001). In contrast, males had significantly lower glucose values at the end of the experiment compared to the beginning in both treatment groups. Low circulating glucose levels could be indicative of depleting energy levels from the fatigue of captivity and increased metabolism during stressful conditions.

Triglycerides in reptiles represent most of the excess energy stores that are known to fuel energetic demands during extended periods of starvation and reproduction (Price, 2017; McCue, 2010). Our results showed that final triglyceride levels of males were significantly higher than the initial values in both the control and stressed treatment groups, suggesting usage of stored lipid reserves to meet increased energetic investments under stressful conditions when glucose reserves are limiting. The pattern seen in males is suggestive of those seen in birds and mammals which show a tight positive-correlation between corticosterone and metabolic rates (Jimeno & Verhulst, 2023). Increased metabolic rates during stress responses, mediated by corticosterone, needs to be supplemented with energy metabolites, such as glucose, or stored lipids when glucose reserves are limiting. Loss in body mass of lizards from both treatment groups is further indicative of the negative effects of high corticosterone levels that could lead to muscle loss in prolonged time frames as in our experimental phase (Nelson, 2005; Sapolsky et al., 2000).

During ecological challenges, animals usually respond by mounting a stress response mediated by glucocorticoids, such as corticosterone, that induces increased energy utilization to combat the challenging period (Sapolsky et al., 2000). When stored energy reserves get utilized and pre-ingestive nutrient acquisition is limited, animals can meet their increased stress-induced energetic demands through post-ingestive regulation of ingested nutrients (Simpson & Raubenheimer, 2012; Behmer, 2009; Hatch & Afik, 1999). Animals are expected to selectively retain carbon-rich macronutrients such as carbohydrates and lipids, which can be rapidly converted for metabolic requirements, and eliminate excess ingested nitrogen-rich proteins which are costly to store if not utilized (Cavigliasso et al., 2020; Simpson & Raubenheimer, 2012; Hawlena & Schmitz, 2010). In our study, we found that lizards from both control and stressed treatment groups ingested and egested similar amounts of carbon and nitrogen, but retained ~93% of ingested carbon and ~71% of ingested nitrogen. This is nearly 1.3 times more carbon retention than nitrogen. Further, lizards from both the control and stressed treatment groups also released similar amounts of urates, which is primary excretory product of protein metabolism in lizards (Minnich, 1972). Given that both males and female lizards from the control and stressed treatment groups had high corticosterone levels at the end of experiment, similar retention patterns in both treatment groups were not surprising. Retention levels were similar between the sexes as well, even though males in the stressed treatment group had the highest elevation in corticosterone levels.

In our study with the ectothermic vertebrate, *Psammophilus dorsalis*, we find no support for post-ingestive elemental retention as a compensatory strategy to meet energetic demands in challenging conditions. We caution against eliminating this process as a possible stress-regulating mechanism because all lizards in our study mounted a corticosterone response, regardless of their treatment group. Ectothermic vertebrates are under growing threat from habitat loss and climate change (Pontes-da-Silva et al., 2018; Deutsch et al., 2008) often leading to resource limitations and foraging activity constraints (Huey & Kingsolver, 2019). When ecological challenges enforce new nutritional demands, animals should not only respond through pre-ingestive strategies but also adjust post-ingestive elemental retention to meet energetic goals and avoid fitness costs (Cavigliasso et al., 2020; Simpson & Raubenheimer, 2012, 1993; Raubenheimer & Simpson, 2003). In fact, selection should favour both strategies to obtain optimal or near-optimal nutrient amounts in response to varying ecological challenges (Cavigliasso et al., 2020). In the end, it is important to note that post-ingestive nutrient regulation can occur either by modulating absorption of nutrients in the gut or through changes in the metabolic fate of the absorbed nutrients (Cavigliasso et al., 2020; Simpson & Raubenheimer, 2012; Clissold et al., 2010). For example, carbohydrates can be either stored in the form of lipids for later use or immediately used up in respiration (Simpson & Raubenheimer, 2012; Zanotto et al., 1997). In our study, similar loss of body mass in lizards from both treatment groups suggests that ingested resources were utilized and not stored. Determining the fate of these absorbed nutrients may hold intriguing insights about nutrient regulation in response to stress which is beyond the scope of the current study but nonetheless warrants future research.

## Supplementary Material

Supplementary Materials

## Figures and Tables

**Table 1:** Weight ingested (g), egested (g), and retention efficiency (values indicate mean ± s.e.m.) of carbon and nitrogen calculated from the experimental phase (cumulative estimates over 10 days) for males and females of *Psammophilus dorsalis* from the control and stressed treatment groups. Statistical comparisons between control and stressed treatment groups are presented as post-hoc t- or z- ratios along with their associated p-values. The global models included treatment and sex as a two-way interaction.

		*Males*				*Females*			
		Control	Stressed	t- or z-ratio*	p-value	Control	Stressed	t- or z-ratio*	p-value
**Carbon**	Ingested (g)	5.625 ± 0.098	5.515 ± 0.124	0.126	0.901	3.078 ± 0.482	2.908 ± 0.343	0.383	0.707
	Egested (g)	0.393 ± 0.014	0.404 ± 0.023	-0.315	0.756	0.219 ± 0.014	0.226 ± 0.019	-0.387	0.704
	Retention efficiency	0.93 ± 0.002	0.926 ± 0.005	0.305	0.761	0.919 ± 0.013	0.921 ± 0.004	0.243	0.808
**Nitrogen**	Ingested (g)	0.828 ± 0.014	0.812 ± 0.018	0.126	0.901	0.453 ± 0.071	0.428 ± 0.051	0.383	0.707
	Egested (g)	0.226 ± 0.011	0.237 ± 0.011	-0.535	0.599	0.121 ± 0.008	0.122 ± 0.009	-0.130	0.898
	Retention efficiency	0.728 ± 0.009	0.707 ± 0.018	0.487	0.626	0.699 ± 0.045	0.704 ± 0.029	0.019	0.985

* t-ratio for ingested and egested, whereas z-ratio for retention efficiency
