# Supplementary material for: Stress–induced elemental retention in an ectothermic vertebrate
Source: Biol Open. Author manuscript; Available in PMC 2025 Oct 15. (PMC7618149; doi:10.1242/bio.062237)
Supplement: Supplementary file 1 [file EMS208788-supplement-Supplementary_Materials.pdf]

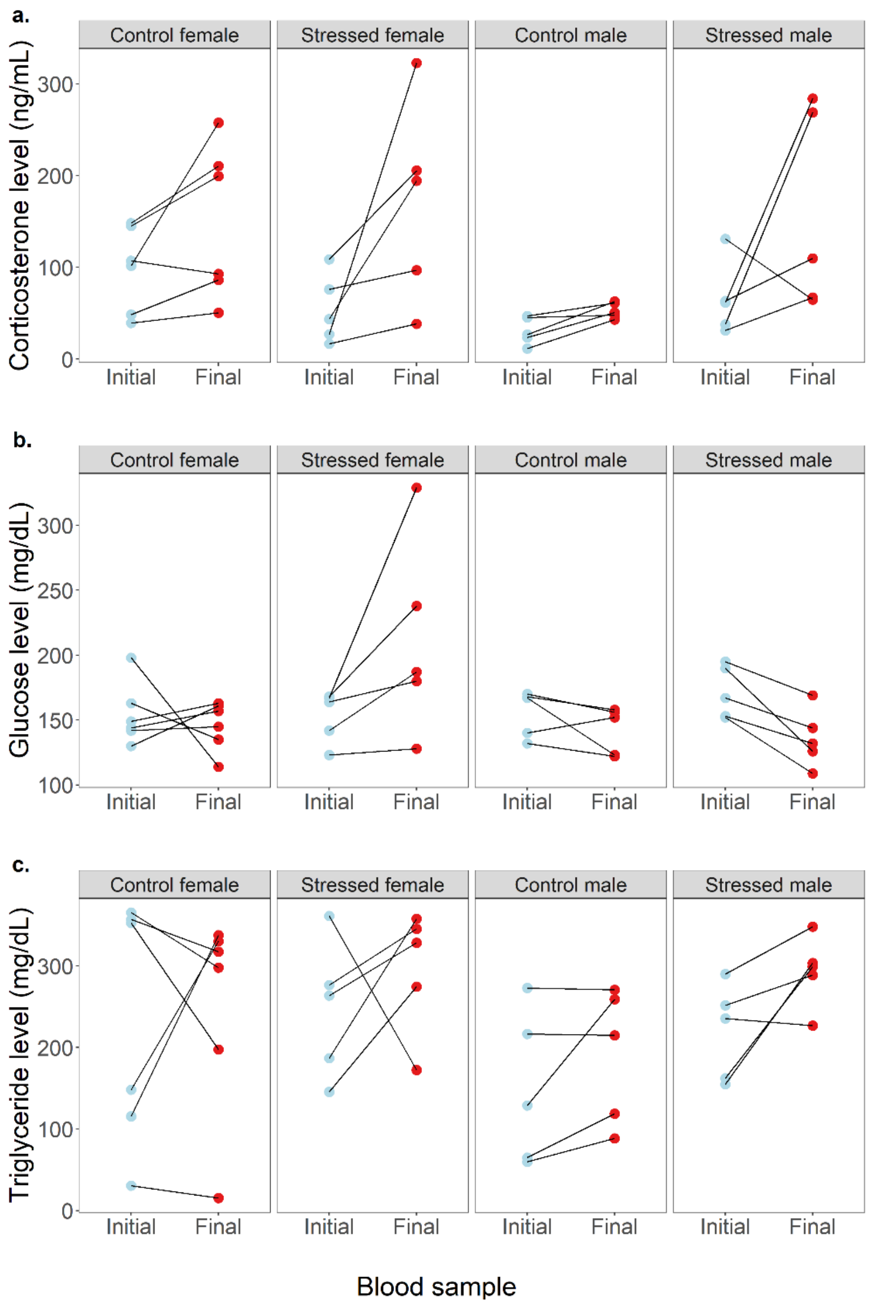

Fig. S1. Initial (blue) and final (red) levels of (a) baseline corticosterone, (b) glucose, and, (c) triglyceride levels in females and males of *Psammophilus dorsalis* from the control and stressed treatment groups. Black solid lines connect the initial and final values for each lizard in the treatment.

Biology Open • Supplementary information

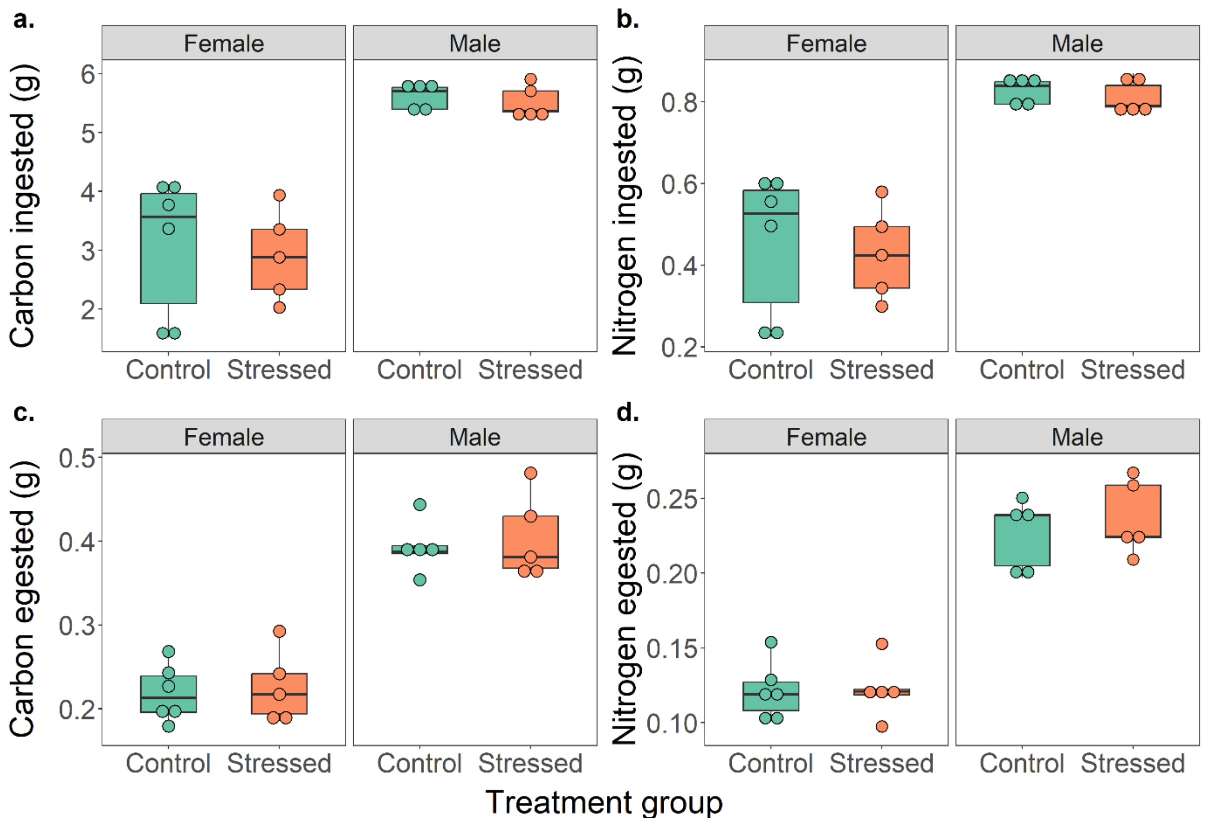

Fig. S2. Weight (g) of ingested (a) carbon and (b) nitrogen and egested (c) carbon and (d) nitrogen in control (green) and stressed (orange) treatment groups of females and males of *Psammophilus dorsalis*.

Biology Open • Supplementary information

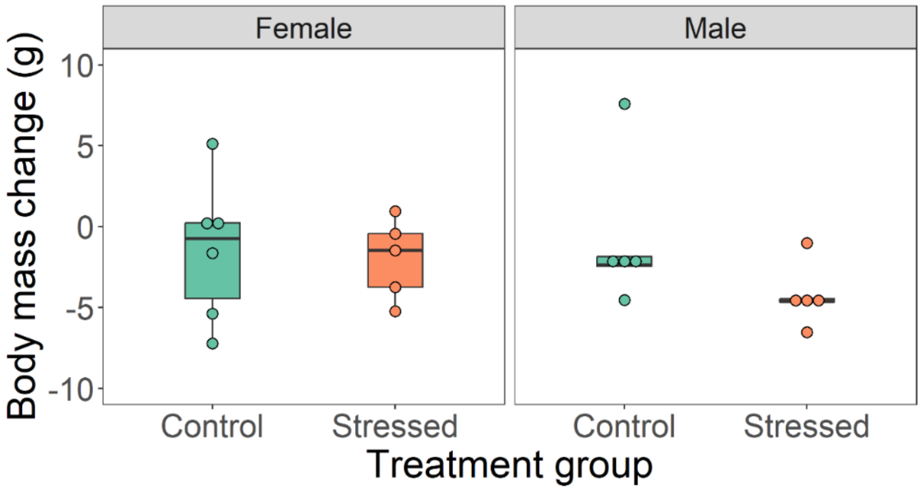

Fig. S3. Change in body mass of females and males of *Psammophilus dorsalis* from the control (green) and stressed (orange) treatment groups. Change in body mass (in grams) was calculated as the difference between final (after treatment) and initial (at capture) mass of lizards.

Biology Open • Supplementary information

Table S1. Coefficients from the models predicting baseline corticosterone level, glucose level, triglyceride level, elemental ingestion, egestion, retention efficiency, and body mass change. Predictors for the corticosterone, glucose, and triglyceride models (sexes separate) include treatment group (control or stressed) and blood collection time (beginning or end of treatment) as predictor variables with animal ID as random variable. Models on carbon and nitrogen ingestion, egestion, retention efficiency and body mass change of lizards include treatment group and sex as the predictor variables.

Parameter ~ Num * Treatment + (1|ID), family= Gamma (link='log')										
**Parameter**			**Estimate**	**SE**	**t-value**	**p-value**		**Random effects**	**Variance**	**Std.Dev.**
Corticosterone levels	Male	(Intercept)	3.965	0.237	16.726	0.000		Animal ID	0.062	0.249
		num_initial	-0.601	0.262	-2.293	0.022				
		treatment_Stressed	1.025	0.342	2.992	0.003				
		num_initial:treatment_Stressed	-0.266	0.379	-0.700	0.484				
	Female	(Intercept)	4.838	0.324	14.936	< 2e-16		Animal ID	0.232	0.482
		num_initial	-0.384	0.187	-2.050	0.040				
		treatment_Stressed	0.105	0.482	0.218	0.828				
		num_initial:treatment_Stressed	-0.737	0.286	-2.580	0.010				
Glucose levels	Male	(Intercept)	4.951	0.069	71.956	<2e-16		Animal ID	0.007	0.085
		num_initial	0.090	0.043	2.068	0.039				
		treatment_Stressed	-0.049	0.097	-0.507	0.613				
		num_initial:treatment_Stressed	0.146	0.061	2.381	0.017				

Biology Open • Supplementary information

	Female	(Intercept)	4.983	0.092	54.255	0.000		Animal ID	0.014	0.116
		num_initial	0.054	0.081	0.674	0.500				
		treatment_Stressed	0.331	0.137	2.426	0.015				
		num_initial:treatment_Stressed	-0.351	0.120	-2.933	0.003				
Triglyceride levels	Male	(Intercept)	5.132	0.241	21.316	0.000		Animal ID	0.098	0.313
		num_initial	-0.331	0.105	-3.147	0.002				
		treatment_Stressed	0.546	0.341	1.602	0.109				
		num_initial:treatment_Stressed	0.020	0.149	0.131	0.896				
	Female	(Intercept)	5.152	0.401	12.835	<2e-16		Animal ID	0.338	0.582
		num_initial	-0.054	0.200	-0.269	0.788				
		treatment_Stressed	0.538	0.596	0.903	0.367				
		num_initial:treatment_Stressed	-0.152	0.293	-0.518	0.604				
Parameter ~ Treatment * Sex, family= Gamma(link = "log")										
**Parameter**			**Estimate**	**SE**	**t-value**	**p-value**				
Carbon Ingested (weight)		(Intercept)	8.032	0.101	79.909	0.000				
		treatment_Stressed	-0.057	0.149	-0.383	0.707				
		sex_male	0.603	0.149	4.043	0.001				
		treatment_Stressed:sex_male	0.037	0.216	0.174	0.864				

Biology Open • Supplementary information

Carbon Egested (weight)		(Intercept)	5.387	0.059	91.391	< 2e-16				
		treatment_Stressed	0.034	0.087	0.387	0.704				
		sex_male	0.586	0.087	6.704	0.000				
		treatment_Stressed:sex_male	-0.005	0.126	-0.040	0.969				
Nitrogen Ingested (weight)		(Intercept)	6.116	0.101	60.850	< 2e-16				
		treatment_Stressed	-0.057	0.149	-0.383	0.707				
		sex_male	0.603	0.149	4.043	0.001				
		treatment_Stressed:sex_male	0.037	0.216	0.174	0.864				
Nitrogen Egested (weight)		(Intercept)	4.797	0.056	86.115	< 2e-16				
		treatment_Stressed	0.011	0.083	0.130	0.898				
		sex_male	0.624	0.083	7.548	0.000				
		treatment_Stressed:sex_male	0.035	0.119	0.297	0.770				
Parameter ~ Treatment , family= beta_family()										
**Parameter**			**Estimate**	**SE**	**z-value**	**p-value**				
Carbon retention efficiency		(Intercept)	2.470	0.091	27.203	<2e-16				
		treatment_Stressed	-0.032	0.133	-0.243	0.808				
		sex_male	0.095	0.137	0.689	0.491				

Biology Open • Supplementary information

		treatment_Stressed:sex_male	-0.012	0.197	-0.060	0.953				
Nitrogen retention efficiency		(Intercept)	0.864	0.121	7.170	0.000				
		treatment_Stressed	-0.003	0.178	-0.019	0.985				
		sex_male	0.098	0.181	0.543	0.587				
		treatment_Stressed:sex_male	-0.088	0.260	-0.341	0.733				
Parameter ~ Treatment * Sex										
**Parameter**			**Estimate**	**SE**	**t-value**	**p-value**				
Body mass difference		(Intercept)	-1.447	1.499	-0.965	0.348				
		treatment_Stressed	-0.533	2.223	-0.240	0.813				
		sex_male	0.727	2.223	0.327	0.748				
		treatment_Stressed:sex_male	-2.999	3.214	-0.933	0.364				

Biology Open • Supplementary information

Table S2. Confidence intervals (95% low and high) generated from bootstrapping model coefficients of the models predicting corticosterone, glucose, and triglyceride levels.

Parameter		Term	Confidence interval_low	Confidence interval_high
Corticosterone level	Male	(Intercept)	3.807	4.111
		num_initial	-1.038	-0.266
		treatment_Stressed	0.349	1.477
		num_initial:treatment_Stressed	-1.052	0.789
	Female	(Intercept)	4.357	5.184
		num_initial	-0.582	-0.146
		treatment_Stressed	-0.577	0.938
		num_initial:treatment_Stressed	-1.587	-0.091
Glucose level	Male	(Intercept)	4.842	5.052
		num_initial	-0.025	0.198
		treatment_Stressed	-0.177	0.065
		num_initial:treatment_Stressed	0.022	0.315
	Female	(Intercept)	4.873	5.055
		num_initial	-0.102	0.289
		treatment_Stressed	0.080	0.632
		num_initial:treatment_Stressed	-0.697	-0.107
Triglyceride level	Male	(Intercept)	4.643	5.601
		num_initial	-0.599	0.008
		treatment_Stressed	0.016	0.993
		num_initial:treatment_Stressed	-0.310	0.372
	Female	(Intercept)	4.530	5.934
		num_initial	-0.761	0.416
		treatment_Stressed	-0.220	1.138
		num_initial:treatment_Stressed	-0.861	0.675

Table S3. Pairwise comparisons between initial and final values from control and stressed treatment groups (within-group) and between control and stressed treatment groups for initial and final levels (between-group) of baseline corticosterone, glucose, and triglyceride levels for males and females of *Psammophilus dorsalis*.

Parameter	Sex	Treatment	Contrast	estimate	SE	z-ratio	p-value
Corticosterone level	Male	Control	Final/Initial	1.820	0.478	2.293	0.022
		Stressed	Final/Initial	2.380	0.647	3.183	0.002
	Female	Control	Final/Initial	1.470	0.275	2.050	0.040
		Stressed	Final/Initial	3.070	0.663	5.191	<.0001
Glucose level	Male	Control	Final/Initial	0.914	0.040	-2.068	0.039
		Stressed	Final/Initial	0.790	0.034	-5.435	<.0001
	Female	Control	Final/Initial	0.947	0.076	-0.674	0.500
		Stressed	Final/Initial	1.345	0.119	3.354	0.001
Triglyceride level	Male	Control	Final/Initial	1.390	0.147	3.147	0.002
		Stressed	Final/Initial	1.370	0.143	2.968	0.003
	Female	Control	Final/Initial	1.060	0.211	0.269	0.788
		Stressed	Final/Initial	1.230	0.263	0.960	0.337
Corticosterone level	Male	Initial	Control/ Stressed	0.468	0.160	-2.226	0.026
		Final	Control/ Stressed	0.359	0.123	-2.992	0.003
	Female	Initial	Control/ Stressed	1.880	0.905	1.315	0.189
		Final	Control/ Stressed	0.900	0.434	-0.218	0.828
Glucose level	Male	Initial	Control/ Stressed	0.908	0.088	-0.995	0.320
		Final	Control/ Stressed	1.051	0.102	0.506	0.613
	Female	Initial	Control/ Stressed	1.019	0.139	0.141	0.888

Biology Open • Supplementary information

		Final	Control/ Stressed	0.718	0.098	-2.426	0.015
Triglyceride level	Male	Initial	Control/ Stressed	0.568	0.194	-1.659	0.097
		Final	Control/ Stressed	0.579	0.197	-1.602	0.109
	Female	Initial	Control/ Stressed	0.680	0.405	-0.649	0.516
		Final	Control/ Stressed	0.584	0.348	-0.903	0.367

Biology Open • Supplementary information

Table S4. Pairwise comparisons between control and stressed treatment groups for amount of carbon and nitrogen ingested, egested, their retention efficiency, and lizards' body mass change for females and males of *Psammophilus dorsalis*.

**Parameter**		**Contrast**	**estimate**	**SE**	**t-ratio**	**p-value**
Carbon Ingested (weight)	Female	Control/Stressed	1.060	0.158	0.383	0.707
	Male	Control/Stressed	1.020	0.159	0.126	0.901
Carbon Egested (weight)	Female	Control/Stressed	0.967	0.085	-0.387	0.704
	Male	Control/Stressed	0.972	0.089	-0.315	0.756
Nitrogen Ingested (weight)	Female	Control/Stressed	1.060	0.158	0.383	0.707
	Male	Control/Stressed	1.020	0.159	0.126	0.901
Nitrogen Egested (weight)	Female	Control/Stressed	0.989	0.082	-0.130	0.898
	Male	Control/Stressed	0.955	0.082	-0.535	0.599
Body mass difference	Female	Control/Stressed	0.533	2.220	0.240	0.813
	Male	Control/Stressed	3.532	2.320	1.521	0.147
**Parameter**		**Contrast**	**estimate**	**SE**	**z-ratio**	**p-value**
Carbon retention efficiency	Female	Control/Stressed	1.030	0.138	0.243	0.808
	Male	Control/Stressed	1.050	0.151	0.305	0.761
Nitrogen retention efficiency	Female	Control/Stressed	1.000	0.179	0.019	0.985
	Male	Control/Stressed	1.100	0.207	0.487	0.626

Dataset 1. Stress-induced elemental retention in an ectothermic vertebrate

Available for download at
https://journals.biologists.com/bio/article-lookup/doi/10.1242/bio.062237#supplementary-data
